# Bioactive-Chemical Quality Markers Revealed: An Integrated Strategy for Quality Control of Chicory

**DOI:** 10.3389/fnut.2022.934176

**Published:** 2022-07-04

**Authors:** Yaolei Li, Shanshan Ju, Zhijian Lin, Hao Wu, Yu Wang, Hongyu Jin, Shuangcheng Ma, Bing Zhang

**Affiliations:** ^1^School of Chinese Pharmacy, Beijing University of Chinese Medicine, Beijing, China; ^2^National Institutes for Food and Drug Control, Beijing, China

**Keywords:** *Cichorium glandulosum* Boiss.et Huet, *Cichorium intybus* L., Quality marker, bioactive-chemical, quality control, network pharmacology

## Abstract

As a miraculous Xinjiang Uyghur customary traditional Chinese medicine (TCM), Chicory (*Cichorium glandulosum* Boiss.et Huet and *Cichorium intybus* L.) has been found to have therapeutic potential for metabolic diseases in recent years. Although it is widely used as an ethnic medicine, there is still a lack of targeted quality control indicators in quality standards. Hence, this study was conducted to further develop a strategy to reveal bioactive-chemical quality markers based on the existing foundation. First, through the comparative screening of fingerprint profiles of a large amount of *Cichorium glandulosum* Boiss.et Huet and *Cichorium intybus* L., superiority components were found to be potential indicators of chemical quantitative properties for the roots and above-ground parts. The results of content determination showed that their contents differed among different species and parts. Second, the potential dominant components were further confirmed using network pharmacology and molecular docking techniques. Again, the results of RAW264.7 cells and L02 cells experiments showed that chicory acid and lactucin were the main components that could reflect the anti-inflammatory and uric acid-lowering potential of chicory. Finally, under this strategy, this study reveals that cichoric acid and lactucin have the properties of quality markers and quality control of chicory. In a word, this work contributes to the quality control, standard improvement, and rational clinical use of chicory.

## Introduction

In recent years, the use of traditional Chinese medicine (TCM) in major public health events, particularly in times of novel coronavirus epidemics, has led to its beneficial role in the treatment of diseases (1–3). As a part of Chinese civilization, the quality of TCM is closely related to its efficacy. With the widespread use of TCMs, their quality control has drawn strong attention from pharmacologists. Academician Changxiao Liu. has proposed the concept of “Quality markers” (Q markers) since 2016 (4, 5), and many researchers have subsequently used it as an entry point for research (6). It is well-known that some components of TCM are more active and are the main medicinal components, for example, artemisinin in *Artemisia annua* L., berberine in *Coptis chinensis* Franch., baicalin in *Scutellaria baicalensis* Georgi, and so on (7–9). However, in many existing studies, some of them only focus on the chemical components in TCM, but ignore the active components that represent the medicinal effects. This has led to one-sided quality standards, which are difficult to connect accurately with the therapeutic effects. Recently, researchers have used chemical composition-bioactivity association characterization for quality control studies of TCMs. For example, (10) used a bioactive-chemical quality marker discovery strategy to characterize *Typha orientalis*. Li’s study has also demonstrated that a similar strategy could be used as an effective approach for discovering the Q marker of *Periplocae Cortex* (11). However, many TCM standards are lagging in research and have difficulty matching new efficacy and new uses. Unreasonable quality standards do not even facilitate resource development and clinical application. In summary, how to reveal the Q markers of TCM in a reasonable way has become a key scientific issue.

Chicory, as an Uyghur customary herb in China, is also known as “kasni” and “kashenna” (12). It is the dried above-ground parts or roots of *Cichorium glandulosum* Boiss. et Huet and *Cichorium intybus* L. of the Asteraceae family (13). Chicory has been introduced to China in the 13th century. It is used as a single herb or in compound formulas. Chicory is effective in clearing the liver and gallbladder, strengthening the stomach, and eliminating food, diuretic, and swelling. Its medicinal properties are slightly bitter, salty, and cool. It is classified as a member of the liver, gall bladder, and stomach meridians (14). The pharmacological activity of chicory and its mechanism of action have been studied intensively in recent years. According to the literature, chicory mainly has anti-inflammatory, antioxidant, anti-tumor, anti-viral, anti-diabetic, anti-obesity, immunomodulatory, and other pharmacological effects (15). Meanwhile, as a medicinal food source, chicory has a wide range of applications in food processing, such as tea and coffee (16–18). *Cichorium intybus* L. is widely distributed in China, both wild and cultivated. The wild product is perennial, and it is more distributed in the Xinjiang region (19). Besides, it is grown in Heilongjiang, Shanxi, Liaoning, and other places. *Cichorium glandulosum* Boiss. et Huet is mainly a cultivated product that is 1 year old and is mainly distributed in the Xinjiang region (20). As a member of the genus Chicory in the family Asteraceae, *Cichorium intybus* L. and *Cichorium glandulosum* Boiss. et Huet are rich in chemical composition, with about 200 chemical components being reported so far (21, 22). It includes phenylpropanoids, flavonoids, sesquiterpenes, triterpenes, steroids, organic acids, alkaloids, and other components. Among them, chlorogenic acid, esculetin, lactucin, cichoric acid, isochlorogenic acid A, 11β,13-dihydrolactucopicrin, and quercetin are the main components reported (23).

Chicory has a high medicinal value. Our group has recently discovered that chicory has new effects on the prevention and treatment of hyperuricemia and gout (24). We conducted comprehensive studies on the efficacy and mechanism of action of chicory extract and its key components in the treatment of the disease at the *in vivo* organ perfusion, cellular level and animal level, respectively (25–27). In terms of pharmacodynamic effects at the animal level, chicory was able to reduce serum uric acid levels in hyperuricemic quail. It also inhibited xanthine oxidase (XOD) and adenosine deaminase (ADA) activities in serum, thus reducing uric acid production (28). Chicory was also able to significantly inhibit GLUT9 protein expression and promote renal uric acid excretion (29). At the organ perfusion and cellular level, our group applied the isolated kidney perfusion technique combined with *in vitro* HKC cells to screen for uric acid-lowering components. Cichoric acid improved renal clearance and significantly inhibited GLUT9 protein expression in HKC cells (30). The above study showed that chicory has significant new efficacy in lowering uric acid.

Chicory is included in the Chinese Pharmacopoeia, 2020 edition, under the item of medicinal materials and tablets. The current quality standard covers the test items of [characterization], [identification], [examination], and [leaching], but there is no content determination item (13). For the current standard, the following problems mainly exist: (i) the quality standard is lagging, and it is difficult to match the current quality requirements. At present, chicory is not only used as a single herb, but also used in Chinese patent medicine. However, all of them lack markers for quality evaluation. (ii) The standard of chicory lacks content control and fingerprint analysis of index components, including different medicinal parts (root and stem). Therefore, it is difficult to evaluate the quality comprehensively. (iii) In addition, it fails to reflect the new efficacy of the main components in chicory. At present, the main components of chicory are more clearly studied, but the components reflecting its medicinal effects are unknown. Therefore, there is an urgent need to establish the discovery strategy of Q markers for standard improvement.

To address the problems of chicory, this study was conducted to further develop a strategy to reveal bioactive-chemical Q markers. Fingerprint profiles and content of multiple batches of *Cichorium glandulosum* Boiss. et Huet and *Cichorium intybus* L. were analyzed. Network pharmacology and molecular docking techniques were further screened for potential dominant components. Cell experiments were performed to verify the main components of the medicinal activity of chicory. This study intends to reveal the key components with Q marker properties in anticipation of rational quality control of chicory.

## Materials and Methods

### Reagents and Materials

The HPLC-grade methanol, acetonitrile, phosphoric acid, and dimethyl sulfoxide (DMSO) were obtained from Fisher (Fisher, United States). Water used for the experiment was produced by a Milli-Q system (Merck Millipore, United States). Reference standards of chlorogenic acid, esculetin, cichoric acid, and quercetin were obtained from National Institutes for Food and Drug Control (Beijing, China), lot numbers 110753-202018, 110741-202109, 111752-201703, and 100081-201610, with a purity of 96.10, 96.00, 97.6, and 99.1%, respectively. Lactucin and 11β,13-dihydrolactucopicrin were purchased from Shanghai Yuanye Biotechnology Co., Ltd. (Shanghai, China), the lot numbers were A09GB144753 and A28GB144754, respectively, with purity ≥ 95%. Isochlorogenic acid A was purchased from Shanghai Constantine Standard Technical Service Co. Ltd. (Shanghai, China), with the lot number STC0680120 and purity ≥ 98%. Allopurinol and xanthine were purchased from Shanghai Yuanye Biotechnology Co. Ltd. (Shanghai, China).

To ensure the extensiveness and representativeness of *Cichorium glandulosum* Boiss. et Huet and *Cichorium intybus* L., this study was collected from various herb markets, pharmacies, herb companies, and main production areas. The samples included above-ground parts and roots. There were 32 batches of above-ground parts of *Cichorium intybus* L. (leaves and stems, numbered JJ1-JJ32), 39 batches of *Cichorium intybus* L. roots (numbered JJG1-JJG39), and 11 batches of above-ground parts of *Cichorium glandulosum* Boiss. et Huet (numbered MJJ1-MJJ15), including control herbs and cultivated products. *Cichorium glandulosum* Boiss. et Huet root. There are 5 batches of *Cichorium glandulosum* Boiss. et Huet roots (Nos. MJJG1-MJJG5). The samples were identified by Professor Yaojun Yang of the Beijing University of Chinese Medicine. It is the dried above-ground part or root of *Cichorium glandulosum* Boiss. et Huet and *Cichorium intybus* L., family Asteraceae. Sample information is provided in Supplementary Table 1. All sample specimens were stored under cool conditions in the laboratory of the Department of Clinical Chinese Medicine, School of Chinese Pharmacy, Beijing University of Chinese Medicine.

The RAW 264.7 cells and L02 cells were purchased from the Cell Bank of the Chinese Academy of Sciences (Shanghai, China). Phosphate-buffered saline (PBS), fetal bovine serum (FBS), and DMEM medium were obtained from Gibco Co., Ltd. (Gibco, United States). Uric acid salts were obtained from Sigma Aldrich (St. Louis, MO, United States).

### Preparation of Standard Solutions and Samples

Reference standards of chlorogenic acid, esculetin, lactucin, cichoric acid, isochlorogenic acid A, 11β,13-dihydrolactucopicrin, and quercetin were weighed precisely and dissolved in 70% methanol. The reference mix solution containing chlorogenic acid (1.90 μg), esculetin (5.43 μg), lactucin (3.57 μg), cichoric acid (1.78 μg), isochlorogenic acid A (4.40 μg), 11β,13-dihydrolactucopicrin (0.52 μg), and quercetin (4.96 μg) per 1 mL of solution was prepared.

Take about 0.25 g of the sample powder (40 mesh), put it into a conical flask with stopper, and add 70% methanol to make up to 25 mL. Make it dense stopper and weigh. The sample was extracted by reflux in a water bath (Beijing Kewei Yongxing Instrument Co., Ltd., China) for 60 min. After cooling, weigh again and make up the lost weight with 70% methanol. Shake to homogenize and filter through a 0.22-μm microporous membrane (Tianjin Jinteng Experimental Equipment Co., Ltd., China), and take the filtrate for analysis.

To determine the uric acid-lowering biological activity and anti-inflammatory property of chemical components, the reference standards were first dissolved in DMSO, before diluting to the desired experimental concentration with a culture medium and using for pharmacokinetic experiments.

### Chromatographic Conditions for Fingerprinting and Content Determination

The high-performance liquid chromatography (HPLC, Waters Co. Milford, MA, e2695, United States) was used for fingerprinting analysis and content determination. A CAPCELL PAK C18 (4. 6 mm × 250 mm, 5 μm) column was used at 30? with a flow rate of 0.9 mL/min. Methanol (A) and 0.2% phosphoric acid solution (B) were used as the mobile phase. A gradient elution (0–5 min, 85% B; 5–10 min, 85–75% B; 10–20 min, 75% B; 20–30 min, 75–65% B; 30–45 min, 65% B; and 45–70 min, 65–35% B) was used. The injection volume was 10 μL. Detection wavelengths in this experiment were 254 and 330 nm, respectively.

### Optimization and Validation of Analytical Methods

The analytical conditions are optimized according to the characteristics of the target components. This includes detection wavelength, mobile phase, gradient elution conditions, and flow rate, and finally, chromatographic conditions are determined in terms of the performance of chromatographic behavior. Then the sample processing conditions were briefly examined. Different extraction solvents and different extraction methods (hot reflux and sonication) were explored separately based on the extraction rates. Under the conditions, the quantitative methods for seven potential components were validated in terms of sensitivity, linearity, stability, precision, reproducibility, and recovery in accordance with the guidelines for the validation of analytical methods of Chinese Pharmacopoeia [Chinese Pharmacopoeia Commission (the fourth part) (13)]. The detection values of S/N ratio 3:1 and 10:1 were used as the limits of detection (LOD) and limits of quantification (LOQ), respectively. The linearity was investigated at different injection volumes (1–50 μL) of the mixed reference solutions. Precision was analyzed six times consecutively with the same concentration as the control. Six sample solutions were prepared repeatedly to examine the reproducibility. Meanwhile, the stability was examined by analyzing the same solution at intervals of 48 h. In addition, six precision-weighed samples were added with appropriate amounts of seven components of the reference standards, and the recovery samples were obtained according to the sample processing method to examine the accuracy.

### Network Pharmacology and Molecular Docking

Based on the results of fingerprinting and content determination, seven target components were imported into the Herb Herbology database^1^ and the Swiss Target Prediction database.^2^ The species was selected as ‘‘Homo sapiens,’’ and all the component targets obtained from the two databases were combined and duplicates were removed to obtain the component targets. The keywords ‘‘hyperuricaemia, gouty arthritis’’ were used in OMIM,^3^ Drugbank,^4^ GeneCards,^5^ and DisGeNET.^6^ The component targets and disease targets were imported into the Venny 2.1 online mapping tool platform for analysis, and the intersection targets were obtained as component-disease key targets. The component-disease key targets were imported into Cytoscape software to construct a “component-disease-target” network for chicory.

The obtained component-disease key targets were imported into the STRING 11.5 database,^7^ and the species ‘‘Homo sapiens’’ was selected for searching to obtain the target interaction network. The target interaction files were imported into Cytoscape 3.7.2 software for visualization and analysis. The key targets obtained above were entered into the DAVID 6.8 database,^8^ and the species was limited to “Homo sapiens.” Gene Ontology (GO) and Kyoto Encyclopedia of Genes and Genomes (KEGG) analyses were performed at *P* ≤ 0.05 and FDR ≤ 0.01 to screen for key pathways associated with the disease.

On the Autodock Tools 1.5.6 and PyMOL 2.4.0 software platforms, key proteins were selected for docking simulation to extract each component. The higher gold fraction indicates that the compound has a larger binding surface to the protein crystal and is more active.

### Cell Assays

For cell culture, RAW 264.7 cells and L02 cells were independently cultured in DMEM medium supplemented with 10% FBS, 100 U/mL penicillin, and 100 μg/mL streptomycin. Cells were cultured in a 5% CO_2_ incubator at 37°C and were used for experiments until the cell growth density was approximately 80%.

The cell viability was analyzed using the Cell Counting Kit-8 (CCK-8, Biorigin Biotechnology, Beijing, China) assay. Cells were inoculated into 96-well plates at 100 μL per well, and cells were incubated for 24 h in an incubator with different concentrations of compounds (25-200 μmol/L), and the supernatant was discarded. The absorbance (OD) of each group was measured at 450 nm with an enzyme marker, and the cell viability (CV) was calculated.

The ELISA test was performed for the detection of TNF-α in RAW264.7 cell supernatant. The RAW264.7 macrophages were inoculated in a 24-well plate, and different concentrations of compounds (final concentrations were determined according to the results of the CCK-8 experiment) were added for 5 h. Monosodium urate (MSU) was added at a final concentration of 150 μg/mL for 24 h. The cell supernatant was collected at 4? and centrifuged at 3,000 rpm for 10 min. The cell supernatant was collected, and the level of TNF-α in the cell supernatant was measured according to the instructions of the mouse TNF-α ELISA kit.

The HPLC was performed for the detection of UA in the L02 cell supernatant. The L02 cells were inoculated in a 24-well plate. In the model group was added 500 μL of DMEM basal medium containing 100 μM xanthine, and in the drug administration group was added solutions of different concentrations of each compound based on the model group. In the normal group, DMEM solution without xanthine and drug solution was added, and three replicate wells were set up in each group and incubated for 24 h. HPLC was used to determine the UA of the cell supernatant and further optimized according to a previous study (24). An Agilent Zorbax SB C18 column (4.6 mm × 250 mm, 5 μm) was used as the chromatographic column. The mobile phase was methanol and 0.5% acetic acid aqueous solution (10:90) with isocratic elution (10 min). The flow rate was 0.8 mL/min, and the column temperature was 30°C (λ = 288 nm); the injection volumes of UA standard and sample were 5 and 20 μL, respectively.

### Statistical Analysis

All data are expressed as the mean ± SD of at least three independent experiments. The statistical analysis was performed by GraphPad PRISM software (version 8.01). Statistical differences were analyzed by ANOVA followed by Dunnett’s *post hoc* test. *P* < 0.05 was considered statistically significant. ChemPattern software (Comayne Technology Co., Ltd., China) was used for chemometric analysis.

## Results and Discussion

### Workflow for Bioactive-Chemical Q Markers Revealed in Chicory

**FIGURE 1:**
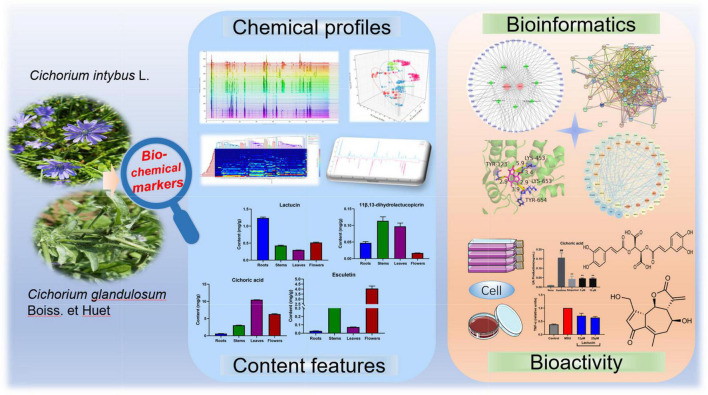
Workflow for bioactive-chemical Q markers revealed in chicory.

Traditional quality control indicators are selected for the major components of the chemical composition, and this simple strategy ignores the manifestation of TCM efficacy. The corresponding limits are set by single chemical composition. Although more directly reflecting the composition and content, this method ignores the characteristics of TCM under the guidance of TCM theory. To address this problem, a screening strategy for biochemical activity-based Q markers of chicory was established in this study (Figure 1). First, the fingerprint profiles of different medicinal parts of multiple species were analyzed with the help of modern analytical tools. The chemical profiles were determined, and the common components were traced to lay the foundation for further analysis. Second, the content determination method was established, and the necessary methodological validation was carried out. Potential components were analyzed to bring out their chemical characteristics by content. Third, after obtaining the target components, we combined network pharmacology and various computer-aided tools to design research methods to establish the correlation between drugs, targets, and diseases from systems biology, multidirectional pharmacology, or network analysis. This rational workflow was utilized to investigate the efficacy and molecular mechanisms of chicory target components for hyperuricemia and gout. Finally, further pharmacodynamics and their mechanism of action were validated using biological cell experiments. In summary, Q markers in chicory were identified. The standard limits of the markers could be assigned by examining multiple batches of samples.

### Optimization and Control of Analysis Methods

In the HPLC method, the combination of methanol and acetonitrile as the organic phase and water, 0.1% formic acid, and 0.2% phosphoric acid as the aqueous phase were optimized, respectively. Based on the peak shape and separation, and with reference to literature reports (31), it was found that methanol was more commonly used compared to acetonitrile. Compared to 0.1% formic acid, 0.2% phosphoric acid as the aqueous phase was more favorable for the separation of multiple complex components in chicory. Finally, methanol and 0.2% phosphoric acid were selected as the mobile phase, and the elution gradient was optimized. Based on the UV maximum absorption by the target components, the final determination was made simultaneously by dual-wavelength method at 254 and 330 nm. The chromatographic separation was better at a flow rate of 0.9 mL/min.

The traditional extraction methods of herbs include ultrasound and heating reflux methods with methanol or ethanol. In this study, ultrasonication and water bath reflux were compared separately, and methanol and 70% methanol were used as extraction solvents, respectively. After fixing the sample sampling amount, time, and solvent, the chromatograms and peak areas were compared by different methods. It was found that hot reflux extraction in a water bath was superior to the ultrasonic method. Moreover, 70% methanol extraction was superior to methanol extraction at the same sampling volume (Supplementary Figure 1). Through the comprehensive comparison, the extraction method of the samples was determined to use 70% methanol in a water bath with reflux extraction for 1 h.

The results of methodological validation showed that the correlation coefficients of chlorogenic acid, esculetin, lactucin, cichoric acid, isochlorogenic acid A, 11β,13-dihydrolactucopicrin, and quercetin were ≥ 0.995, and the linearity was good in the linear range. The precision, reproducibility, stability, and recovery were as presented in Table 1, which met the requirements of the analytical methods in Chinese Pharmacopoeia. In addition, the durability of the method was also investigated. The acceptable differences in the results determined by different instruments and columns indicated that the method was durable.

**TABLE 1:** Calibration curves, repeatability, precision, stability, and recovery results of seven analytes in chicory by HPLC.

Analyte	Calibration curves	*R* ^2^	Linear range (μg)	Repeatability (*n* = 6)		Precision RSD (%) (*n* = 6)	Stability RSD (%) (*n* = 6)	Recovery (%) (*n* = 6)		LOD (ng)	LOQ (ng)
				Content (μg/g)	RSD (%)			Average	RSD (%)		
Chlorogenic acid	*y* = 1786.9x − 160.74	1.0000	0.010–0.10	106.6	4.9	1.9	3.7	100.6	1.44	3.57	7.14
Esculetin	*y* = 7440.7x + 3582	0.9951	0.027–0.27	694.0	2.5	0.5	0.9	100	0.87	1.78	3.55
Lactucin	*y* = 10003x + 5704.9	0.9997	0.018–0.18	397.7	2.7	1.8	0.7	97.9	0.58	4.40	8.80
Cichoric acid	*y* = 1823.1x + 438.17	0.9999	0.009–0.09	193.0	5.1	2.2	3.2	102.5	2.47	5.43	10.85
Isochlorogenic acid A	*y* = 3970x + 6457.9	0.9981	0.022–0.22	190.2	3.6	1.3	1.9	99	2.22	0.52	1.05
11β,13-Dihydrolactucopicrin	*y* = 3364.9x − 1368.2	0.9995	0.003–0.03	50.3	5.9	0.4	3.0	97.2	1.17	1.90	3.81
Quercetin	*y* = 1824x − 253.76	0.9999	0.008–0.08	1454.4	1.3	1.4	0.7	93.1	4.11	4.96	9.92
											

### Chemical Profile of Different Parts in Chicory

**FIGURE 2:**
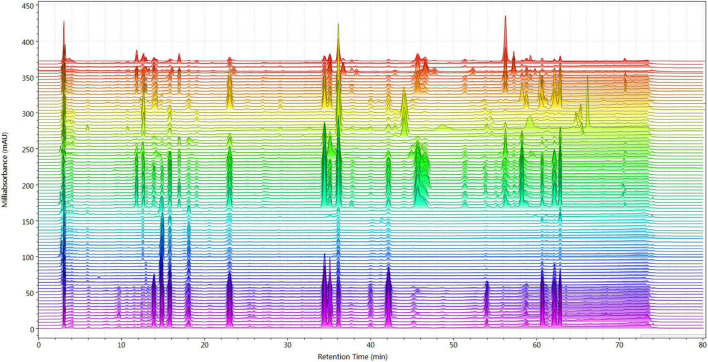
Chromatographic profile of chicory (above-ground parts and roots).

All the chicory samples were first analyzed by fingerprinting according to the established method. As shown in Figure 2, the chromatographic profiles of the medicinal parts of chicory and hairy chicory roots were different. At the detection wavelength of 254 nm, we found that there were more peaks in the samples, which could provide richer fingerprint information and truly reflect the differences between the samples. Therefore, at this wavelength, we analyzed the fingerprint profiles in further detail with the origin of the samples.

**FIGURE 3:**
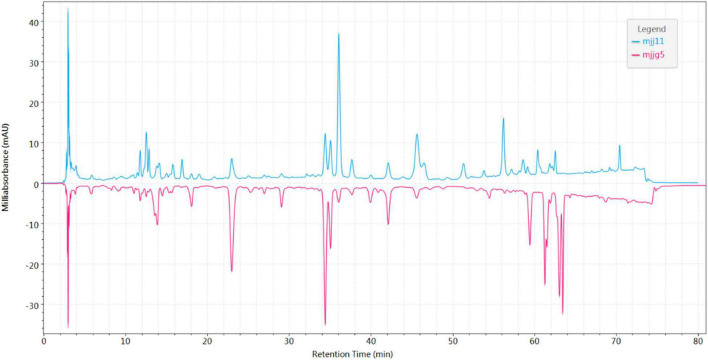
Mirror comparison chromatogram of above-ground parts and roots in *Cichorium glandulosum* Boiss. et Huet.

In *Cichorium glandulosum* Boiss. et Huet, there were slight differences between above-ground and root components. As shown in Figure 3, the content ratios of most components were not consistent in the above-ground parts and roots. *Cichorium glandulosum* Boiss. et Huet is mainly cultivated and usually 1-year-old, and its resources are concentrated in Xinjiang. Unlike *Cichorium glandulosum* Boiss. et Huet, *Cichorium intybus* L. is a perennial plant. Some samples of chicory are obtained from the medicinal herb market as cultivated products, and some other samples are from the main producing area of Xinjiang. In China, *Cichorium intybus* L. cultivation is mainly distributed throughout the country and is used medicinally as well as in food. For the above-ground parts, *Cichorium intybus* L. collected in the main production areas is mainly divided into stems and roots, with fewer leaves. The fingerprint profiles of the above two samples differed significantly, which was found to be different from other herbs in this study (Supplementary Figure 2). The chromatographic information in the stems was rich compared to that obtained from leaves. The *Cichorium intybus* L. root samples were sourced from the market and the main production area, and we found that there were differences in their morphology, with the roots from the main production area being slender in appearance and mostly wild. The roots purchased from the market were thicker in appearance, and most of them were concocted in a flaky form. The comparison of their chromatograms revealed a large difference (Supplementary Figure 3), with the market roots having significantly less chromatographic information than the chicory roots from the production area. Similarly, this study conducted a longitudinal comparison and initially found that the chromatograms of the above-ground parts and roots (origin) of *Cichorium glandulosum* Boiss. et Huet and *Cichorium intybus* L. were similar (Supplementary Figure 4). In summary, the preliminary fingerprint chromatographic profile analysis of multiple batches of samples in this study indicated that there were differences in the chemical composition of *Cichorium glandulosum* Boiss. et Huet and *Cichorium intybus* L. Chemometrics with high efficiency and rapidity were used to analyze the differences in chicory, such as similarity, principal component analysis (PCA), orthogonal partial least squares (OPLS), cluster analysis (CA), etc.

Similarity evaluation was able to show the initial identification of differences between medicinal parts. The chromatographic results were imported into ChemPattern software, and the similarity calculation function was used for fingerprint similarity analysis. In order to carry out the differences between different species and different parts horizontally, the above-ground part of JJ was used as a reference. The distance value is the overall similarity of the samples after unification. The fingerprint chromatographic similarity results visually showed that the comparative analysis of all samples with *Cichorium glandulosum* Boiss. et Huet root as a common pattern revealed (Supplementary Figure 5) that large differences existed between samples with lot numbers MJJ3-MJJ5 in the above-ground part of *Cichorium glandulosum* Boiss. et Huet and other samples. In *Cichorium intybus* L., there were differences in the similarity of leaves (JJ1-JJ16), while the similarity in lots JJ17-JJ32 was more consistent. In *Cichorium intybus* L. root, similarity behavior showed two phenomena: JJG1-JJG25 similarity was close and JJG26-JJG39 similarity was more consistent, while these two parts of roots were market samples and production area samples.

**FIGURE 4:**
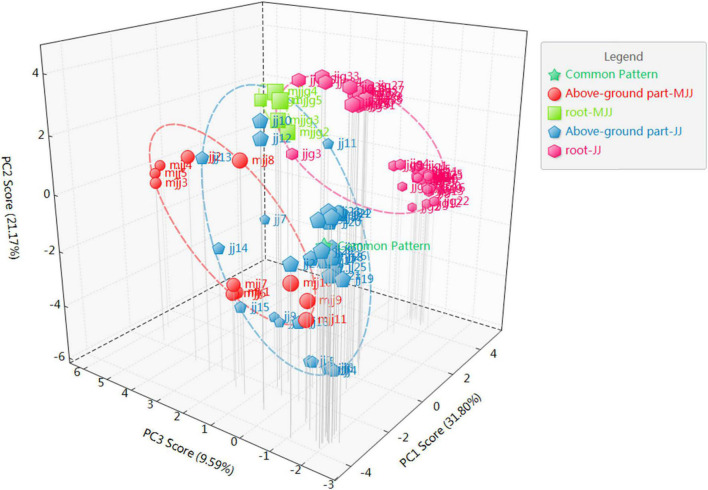
PCA analysis of different medicinal parts (above-ground parts and roots) of chicory.

**FIGURE 5:**
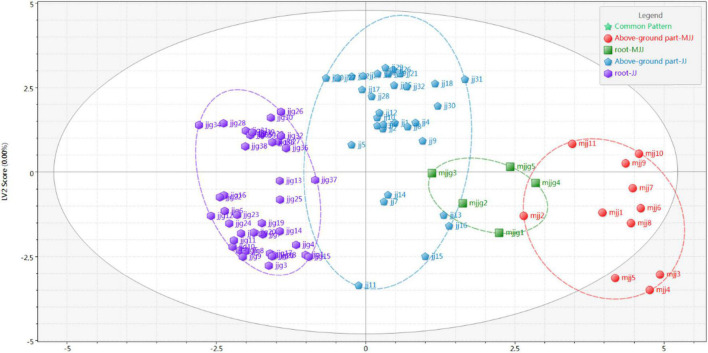
OPLS analysis of different medicinal parts (above-ground parts and roots) of chicory.

The PCA is the most widely used unsupervised pattern recognition method. To visualize the overall distribution of the samples, PCA analysis was performed on all batches of samples in this study (Figure 4). The results showed that the cumulative contribution of the first three principal components was 62.49%, with the first, second, and third principal components contributing 31.80, 21.1, and 9.59%, respectively. Its can characterize most of the extracted chemical composition information. We found that chicory roots could be clearly divided into two groups with close scattering distances, which presented consistent results with the similarity analysis. However, the above-ground part of *Cichorium intybus* L., and the root and above-ground parts of *Cichorium glandulosum* Boiss. et Huet were all indistinguishable in PCA, and the scatter spatial distances were interleaved. Meanwhile, we tried to analyze with OPLS (Figure 5) and found that the four parts of the samples could be basically distinguished.

**FIGURE 6:**
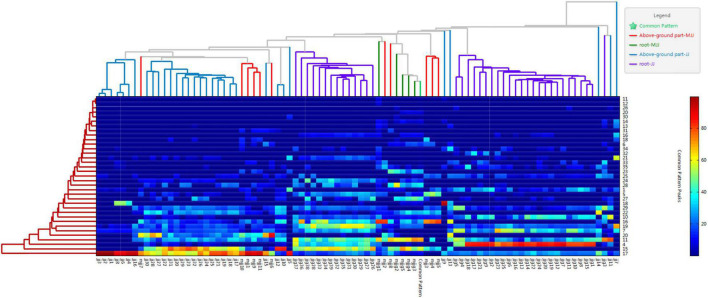
Heat maps of different medicinal parts (above-ground parts and roots) of chicory.

A heat map can filter, extract, and downscale the data, and can reflect the relationship between samples by horizontal clustering and between chemical components by vertical clustering. Therefore, in this study, heat map clustering analysis was performed for all batches of samples. As can be seen from Figure 6, all batches of samples were cluster diversified, but the overall trend was basically consistent with the results of fingerprint profiling. Among them, the leaves of the above-ground part of *Cichorium intybus* L. could be well clustered into one class, and the stems could be obviously clustered into one class. The roots were obviously clustered into two classes, again in agreement with the results of a previous analysis. However, in *Cichorium glandulosum* Boiss. et Huet, the clustering results were more discrete, which might be related to the small number of samples.

In conclusion, we found that *Cichorium intybus* L. and *Cichorium glandulosum* Boiss. et Huet are different through fingerprinting combined with chemometric identification analysis. This difference originated from above-ground parts and roots. In addition, we found that the differences between step-wise varieties were closely related to the source of the samples. Therefore, it is necessary to further characterize the different medicinal parts by content analysis of the target components.

### Multiple Component Content in Different Parts of Chicory

**FIGURE 7:**
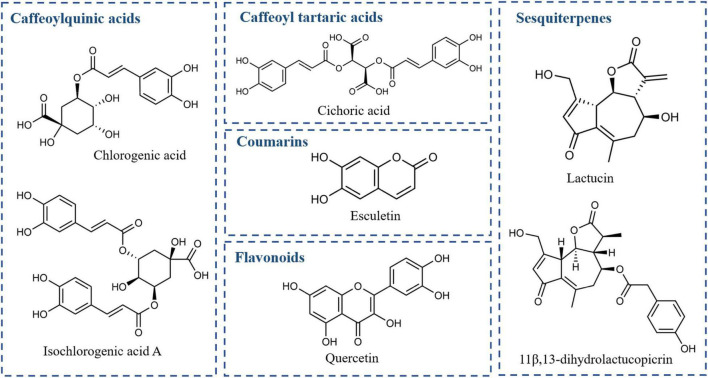
Structures of seven potential Q markers in chicory.

Chicory is rich in chemical constituents. In this study, based on the reports on the chemical constituents of chicory and the available known standards, we selected seven target components of chicory, including chlorogenic acid, esculetin, lactucin, cichoric acid, isochlorogenic acid A, 11β,13- dihydrolactucopicrin, and quercetin, and analyzed as seven target components. The structures of the compounds are shown in Figure 7. Among them, chlorogenic acid and isochlorogenic acid A are the representative components of caffeoylquinic acid, esculetin is a representative component of coumarins, lactucin and 11β,13-dihydrolactucopicrin are representative components of sesquiterpenes, and quercetin is a representative component of flavonoids. Quercetin is a representative component of flavonoids. The UV maximum absorption characteristics of the above seven chemical components were compared and finally determined by the dual-wavelength method at 254 and 330 nm simultaneously. Among them, lactucin and 11β,13-dihydrolactucopicrin showed a higher response at 254 nm, while chlorogenic acid, esculetin, cichoric acid, isochlorogenic acid A, and quercetin showed a higher response at 330 nm.

Multiple samples were screened using the content determination method established in this study. According to the results (Table 2), the mean values of the seven components, chlorogenic acid, esculetin, lactucin, cichoric acid, isochlorogenic acid A, 11β,13-dihydrolactucopicrin, and quercetin, in the above-ground part of *Cichorium intybus* L. were 0.90, 0.32, 0.45, 2.74, 0.71, 0.15, and 0.030 mg/g, respectively. Among them, chicory acid was the main component with higher content. The results regarding the content of the seven components of *Cichorium intybus* L. root samples are presented in Table 3. The mean values of chlorogenic acid, esculetin, lactucin, cichoric acid, isochlorogenic acid A, 11β,13-dihydrolactucopicrin, and quercetin content were 1.17, 0.042, 0.55, 0.32, 1.24, 0.14, and 0.041 mg/g, respectively. The highest content of these components was isochlorogenic acid A, followed by chlorogenic acid. There were differences in the contents of seven components in the above-ground parts and roots of *Cichorium glandulosum* Boiss. et Huet, as presented in Table 4, in which the above-ground parts had the highest chicoric acid content, and the mean values of chlorogenic acid, esculetin, lactucin, cichoric acid, isochlorogenic acid A, 11β,13- dihydrolactucopicrin, and quercetin contents were 0.39, 0.35, 0.27, 1.23, 0.23, 0.03, and 0.04 mg/g, respectively. The mean values of chlorogenic acid, esculetin, lactucin, cichoric acid, isochlorogenic acid A, 11β,13-dihydrolactucopicrin, and quercetin contents were 0.12, 0.02, 1.06, 0.19, 0.65, 0.35, and 0.06 mg/g, respectively. The above-ground part of *Cichorium intybus* L. and *Cichorium glandulosum* Boiss. et Huet had a higher content of chicoric acid, and the root had a higher content of lactucin.

**TABLE 2:** Content characteristics of seven compounds in above-ground parts of *Cichorium intybus* L. (mean ± SD, μg/g).

Sample NO.	Chlorogenic acid	Esculetin	Lactucin	Cichoric acid	Isochlorogenic acid A	11β,13-Dihydrolactucopicrin	Quercetin
JJ1	887.5 ± 27.8	67.4 ± 0.4	22.6 ± 2.1	5447.2 ± 176.4	639.6 ± 26.7	4.5 ± 0.5	16.4 ± 1.3
JJ2	534.1 ± 10.8	102 ± 3.1	24.3 ± 0.7	3332.4 ± 67.2	193.4 ± 5.7	15 ± 1.8	16.8 ± 4.2
JJ3	457.7 ± 3.4	87.8 ± 2.8	24.3 ± 3.5	2874.5 ± 63	179.1 ± 2.6	5.2 ± 1	23 ± 2.5
JJ4	1332.2 ± 8.5	137.4 ± 1.8	29.4 ± 0.6	8310.8 ± 115.2	518.4 ± 6.2	12.3 ± 0.4	36.4 ± 8.8
JJ5	59.8 ± 2.5	24.4 ± 2.7	8.6 ± 1	396.1 ± 7	38.1 ± 3.5	22.5 ± 1.3	19.3 ± 2.7
JJ6	691.1 ± 10.6	48.6 ± 2.3	10.9 ± 2.1	4037.9 ± 68.5	435.7 ± 9	3.7 ± 0.2	31 ± 2.1
JJ7	382.6 ± 15.4	90.1 ± 3.3	7.4 ± 0.8	73.2 ± 6.8	836.3 ± 42.7	3.9 ± 2.5	25.9 ± 1.1
JJ8	779.3 ± 24.5	82.3 ± 3.8	13.1 ± 0.5	4752.8 ± 151.3	410.6 ± 15.3	11.8 ± 0.2	22.2 ± 8.3
JJ9	113.5 ± 6.5	33.3 ± 1.9	9.5 ± 1.3	650.6 ± 41.4	32.5 ± 7	7.6 ± 1.4	37.8 ± 13.8
JJ10	232.7 ± 6.1	335.7 ± 1.7	425.9 ± 5.8	206.3 ± 3.1	174.5 ± 14.4	72.3 ± 2.2	9.5 ± 0.8
JJ11	44.1 ± 1.5	20.6 ± 0.9	336.1 ± 2.5	33.9 ± 1.9	30 ± 1.5	33.9 ± 0.1	20.7 ± 1.2
JJ12	365.3 ± 2.7	725.3 ± 4.7	483.1 ± 3	370.3 ± 6.8	238.1 ± 10.3	87.6 ± 1.7	32.3 ± 6.3
JJ13	193.1 ± 2.8	34.3 ± 3.5	162.6 ± 6	126.1 ± 5.4	98.2 ± 3.6	7.1 ± 0.7	42 ± 2.8
JJ14	185.4 ± 6.7	95.1 ± 0.7	80.8 ± 4.8	91.5 ± 2.3	75.7 ± 2.6	35.5 ± 0.2	25.9 ± 1.9
JJ15	497.8 ± 19	547.6 ± 11.5	164 ± 4.9	1681 ± 28.4	246 ± 8.4	42 ± 1.5	120.8 ± 7.2
JJ16	886.9 ± 23.1	383.7 ± 8.9	374.4 ± 2.7	1837.9 ± 56.1	1101.3 ± 67.4	17.4 ± 1.8	30.8 ± 1.8
JJ17	971.4 ± 20.4	557.8 ± 3	837.9 ± 13.9	2814.1 ± 42.2	628 ± 22.5	268.8 ± 5	17.6 ± 3.3
JJ18	1376.8 ± 9.9	425.4 ± 13.3	1052.5 ± 8.2	3696.1 ± 87.4	922.3 ± 16.9	329.6 ± 4.1	17.6 ± 0.6
JJ19	1898.7 ± 29.8	348.3 ± 4.3	660.5 ± 10.4	4268.5 ± 64.5	1312.1 ± 19.5	381.1 ± 9.9	41.6 ± 2.1
JJ20	1297.5 ± 51.4	282.2 ± 5.7	817.3 ± 55.2	2926.2 ± 176.7	1132.4 ± 19.8	182.7 ± 23.5	31.8 ± 0.2
JJ21	1577.1 ± 29	320.5 ± 5	560.9 ± 19.7	3866.8 ± 63	1308.1 ± 25.3	208.7 ± 12.1	14.9 ± 0.8
JJ22	1367.2 ± 78.1	195.1 ± 8.6	623.8 ± 21.1	2590.3 ± 97.7	1537.6 ± 55.2	301 ± 16.4	29.2 ± 1.1
JJ23	1521.4 ± 53.6	238.9 ± 8.5	982.1 ± 34	3098 ± 77.5	1155.7 ± 38.3	374.8 ± 12.6	21.5 ± 7.8
JJ24	1195.3 ± 33.6	171.4 ± 2	713.1 ± 12.2	2642.1 ± 89.2	1124.7 ± 40	290.8 ± 10.3	32.9 ± 0.9
JJ25	1105.7 ± 40	336.5 ± 9.2	557.1 ± 13.3	2851 ± 58.9	790 ± 9.5	59.5 ± 3.2	24 ± 0.9
JJ26	1951.4 ± 61.8	895.9 ± 11.4	1036.6 ± 27.5	5766.3 ± 156.7	1520.3 ± 29.3	439 ± 16	22.3 ± 18.8
JJ27	1171 ± 150.5	685.8 ± 50.6	360.2 ± 17.7	3640.8 ± 528.3	809 ± 108.5	238.4 ± 13	13.6 ± 2.9
JJ28	1631.7 ± 83.7	548.3 ± 25.9	1044.1 ± 16.7	4411.5 ± 241.2	1996 ± 119	180.1 ± 87.6	103.5 ± 6.5
JJ29	1259.5 ± 61.6	1063.6 ± 33.9	686.2 ± 13.2	4123.4 ± 210.2	806.1 ± 39.9	414.1 ± 13.3	9.7 ± 1.6
JJ30	860.3 ± 24.7	270.4 ± 2.5	506 ± 6.1	1765.1 ± 37.7	681.7 ± 29.4	113.5 ± 3.8	13.3 ± 1.8
JJ31	453 ± 163.3	255.4 ± 45.1	704.9 ± 27.9	1667.3 ± 656.8	466.5 ± 168.9	233.7 ± 6	9.7 ± 1.4
JJ32	1600.6 ± 146.6	904.6 ± 81.2	1109.3 ± 33.9	3417.3 ± 86.1	1175.8 ± 35.7	562 ± 36.6	42.7 ± 9.1

**TABLE 3:** Content characteristics of seven compounds in roots of *Cichorium intybus* L. (mean ± SD, μg/g).

Sample NO.	Chlorogenic acid	Esculetin	Lactucin	Cichoric acid	Isochlorogenic acid A	11β,13-Dihydrolactucopicrin	Quercetin
JJG1	529.7 ± 27.5	26.9 ± 2.2	290.4 ± 8.7	32.6 ± 2	170.1 ± 12.1	8.3 ± 0.3	16.9 ± 2.7
JJG2	432.2 ± 22.7	25.1 ± 2.7	47.1 ± 8.1	27.6 ± 3.7	34.3 ± 1.5	4.9 ± 0.3	5.1 ± 1
JJG3	22.8 ± 3.1	4.9 ± 0.5	258.4 ± 2.8	19 ± 1.3	54.7 ± 0.5	15.2 ± 0.1	0 ± 0
JJG4	509.4 ± 333.3	8.3 ± 2.5	281.4 ± 11.7	27.2 ± 5.3	172.7 ± 81.7	16.6 ± 0.9	9.4 ± 3.5
JJG5	915.2 ± 33.6	12.9 ± 0.2	350 ± 17.7	22.5 ± 3.5	244 ± 5.9	14.8 ± 0.3	11.6 ± 1.9
JJG6	1076.2 ± 34.7	21.4 ± 5	182.1 ± 5.7	28.9 ± 0.8	542.7 ± 15.7	8 ± 0.2	18.3 ± 0.6
JJG7	497.1 ± 23	27.1 ± 3.1	166.2 ± 3.7	19.5 ± 2.2	112.2 ± 6	8.7 ± 1.2	12.4 ± 1
JJG8	679.5 ± 31.4	36.5 ± 2.2	228 ± 5.4	33.7 ± 3.4	167 ± 11.7	15.2 ± 0.5	16.6 ± 3.3
JJG9	572.4 ± 34.2	26.3 ± 2.4	185.3 ± 6	21.5 ± 3.7	125.7 ± 14.6	8.6 ± 0.5	14.3 ± 0.7
JJG10	1100.5 ± 28.9	48.8 ± 0.6	237.8 ± 15.3	42.9 ± 3	199 ± 20.2	11 ± 1	14.1 ± 4.8
JJG11	835.7 ± 64.2	22.4 ± 2.5	208.4 ± 1.3	30.1 ± 5.7	239.4 ± 29.4	8 ± 0.1	10.9 ± 1
JJG12	739.9 ± 18.3	41.8 ± 1.7	159 ± 4.9	22.8 ± 1.6	115.2 ± 6	8.7 ± 0.6	9 ± 2.6
JJG13	935.7 ± 53.6	17.8 ± 6.1	32.3 ± 0.6	31.4 ± 3.1	238.9 ± 27.3	5.4 ± 0.2	9.9 ± 1.2
JJG14	440 ± 6.8	28 ± 0.5	252.9 ± 6.7	17.5 ± 1.7	77.1 ± 7.9	12.1 ± 0.5	9.6 ± 2.6
JJG15	532.4 ± 41.2	31.1 ± 2.5	307.7 ± 20	29.8 ± 5	174.6 ± 17	10.4 ± 0.7	11.2 ± 0.9
JJG16	1491.7 ± 55	82.2 ± 3.3	128.7 ± 7.2	67.9 ± 8.4	235.9 ± 12.3	10.3 ± 0.7	11.8 ± 5.6
JJG17	593.6 ± 14.3	31.7 ± 1	237.2 ± 2.9	29.7 ± 3.1	189.4 ± 7.7	13.6 ± 2.8	5.4 ± 0.7
JJG18	197.6 ± 7.1	5.3 ± 0.1	114.9 ± 6.2	9.1 ± 0.3	23.2 ± 1.6	21.8 ± 0.4	3 ± 0.2
JJG19	963.3 ± 16.3	44.1 ± 1.4	256.7 ± 3.9	40.3 ± 1.2	104.5 ± 6.6	49.1 ± 2.5	0.6 ± 0.9
JJG20	693.9 ± 33.4	35.3 ± 1.8	209.7 ± 3.2	42.7 ± 2.1	163.4 ± 12.9	30.8 ± 1.1	< *LOD*
JJG21	229.1 ± 12.6	4.4 ± 0.2	129 ± 3.6	14.7 ± 0.4	22.6 ± 2.1	23.3 ± 2.1	< *LOD*
JJG22	1254.4 ± 15.7	52.7 ± 0.9	162.9 ± 1.8	47.7 ± 3.4	205.6 ± 10.2	39.7 ± 1.8	< *LOD*
JJG23	1112.5 ± 29.8	38.2 ± 1.6	166.2 ± 4.4	49.3 ± 3.9	315.2 ± 12.5	33 ± 1.3	< *LOD*
JJG24	650.2 ± 21.8	35.3 ± 2.3	130.8 ± 5.6	30.1 ± 1.5	158 ± 6.8	21.2 ± 1.1	< *LOD*
JJG25	1110.6 ± 22.2	23.6 ± 7.8	214 ± 3.8	51.6 ± 2.2	434.5 ± 24	38.5 ± 2.4	< *LOD*
JJG26	1505 ± 58.2	41.7 ± 0.7	992.5 ± 2.5	1039.2 ± 35.8	2513.1 ± 115.4	518 ± 10.8	151.3 ± 6.4
JJG27	2324.3 ± 82.4	79 ± 2.6	1101.7 ± 59.9	584.9 ± 15	2905.4 ± 62.9	300.1 ± 19.4	134.8 ± 6.3
JJG28	2612.3 ± 26.3	91.5 ± 1.5	851.2 ± 13.8	761.5 ± 20.4	3422.6 ± 51.1	351.8 ± 11.7	120.6 ± 57.1
JJG29	2514.5 ± 48.1	103.7 ± 1.8	1097.4 ± 26.6	1045.2 ± 20.1	3725.6 ± 41.3	338.6 ± 9.2	98.1 ± 42.3
JJG30	1872.3 ± 56.7	75 ± 3.9	1051.5 ± 26.5	1294.4 ± 78	3658.9 ± 117.1	418 ± 13.8	156.5 ± 1.9
JJG31	2009.4 ± 164.1	92.5 ± 7.8	893.5 ± 12	842.2 ± 104.1	2860.6 ± 288.9	266.9 ± 8	48.5 ± 44.5
JJG32	2343.9 ± 24.5	94.8 ± 3.9	1559.3 ± 51	1170.8 ± 32.9	3801.5 ± 35.5	147.3 ± 5.1	129.3 ± 8.3
JJG33	1919.6 ± 61.1	64.1 ± 3.3	1487.9 ± 43.3	692.8 ± 43.9	3481.5 ± 168.4	489.8 ± 20.3	88.6 ± 11
JJG34	2431.9 ± 87.6	74 ± 4.5	1096.5 ± 55.6	759 ± 36.3	3571.1 ± 113.8	532.3 ± 28.1	59.3 ± 1.3
JJG35	2477.2 ± 137.2	71.9 ± 3.9	1305 ± 44	979.7 ± 46	3817.6 ± 210.4	170.5 ± 91.2	155.3 ± 10.2
JJG36	1505 ± 286.5	38.4 ± 7.2	1752.9 ± 23.9	768.9 ± 141.6	3947.2 ± 774.2	456.4 ± 14	77.4 ± 34.6
JJG37	1024.3 ± 164.7	17.9 ± 5.8	1345.2 ± 15.8	547.2 ± 95.2	1144.9 ± 213.1	286.4 ± 11	14.6 ± 1.3
JJG38	1154.5 ± 128	38 ± 2.8	721.1 ± 65.5	481.7 ± 46.2	1639.2 ± 197.2	299.3 ± 46	45.9 ± 7.3
JJG39	1807.5 ± 122.7	47 ± 2	1184.9 ± 22	837 ± 48.7	3182.4 ± 233.2	421.1 ± 16.1	102.4 ± 8.8

**TABLE 4:** Content characteristics of seven compounds in *Cichorium glandulosum* Boiss.et Huet (mean ± SD, μg/g).

Sample NO.	Chlorogenic acid	Esculetin	Lactucin	Cichoric acid	Isochlorogenic acid A	11β,13-Dihydrolactucopicrin	Quercetin
MJJ1	355.6 ± 12.7	775.1 ± 4.1	239.2 ± 6.6	2051.5 ± 52.9	130.9 ± 5.3	66.2 ± 5.6	146.1 ± 9
MJJ2	151.2 ± 5.9	25.4 ± 2.9	283.5 ± 4.6	231.1 ± 13	180.1 ± 10.8	17.9 ± 0.4	29.3 ± 12.2
MJJ3	726.6 ± 7.6	659.7 ± 26.9	140.9 ± 1.9	1180.5 ± 39.1	400.6 ± 2.2	18.8 ± 0.7	20.1 ± 0.8
MJJ4	580.2 ± 22.5	386 ± 16.3	237.8 ± 4.3	1323.4 ± 35.2	302.2 ± 7.4	18.8 ± 1.8	37.4 ± 25.4
MJJ5	160.1 ± 1.5	265.7 ± 2.8	381.7 ± 5.3	306.1 ± 4.3	121.4 ± 2	22.8 ± 2	47.1 ± 7.3
MJJ6	371.2 ± 1.4	375.3 ± 1.7	595.6 ± 2.1	2633.3 ± 46.2	567 ± 5	59.7 ± 27.3	13.2 ± 0.1
MJJ7	222.3 ± 0.6	249.9 ± 1.1	272 ± 0.6	1325.3 ± 18.3	273.5 ± 4.5	35.7 ± 5.7	37.5 ± 19.2
MJJ8	234.7 ± 0.6	269.5 ± 1.2	207.8 ± 2.2	1999.9 ± 36.8	360.6 ± 1.5	25.5 ± 4.6	26.5 ± 1.3
MJJ9	449.9 ± 9.3	177.7 ± 3.6	249.4 ± 8.3	651 ± 9.8	52.6 ± 2.1	39.9 ± 2.4	40.9 ± 1.7
MJJ10	485.2 ± 5.1	404.4 ± 4.1	194.6 ± 0.9	878 ± 17.3	46.8 ± 1.3	21.9 ± 0.7	38 ± 0.6
MJJ11	591.2 ± 27	310.7 ± 5.7	191.3 ± 11	967.5 ± 25.2	50.1 ± 0.3	20.6 ± 1	34.7 ± 2.4
MJJG1	75.1 ± 5.7	3.5 ± 0.3	339.5 ± 14.8	84.1 ± 5.2	298.2 ± 15.4	20 ± 1.2	14.5 ± 7.4
MJJG2	96.2 ± 0.6	44.5 ± 1.5	684.9 ± 4	294.7 ± 3.8	144.7 ± 2.3	225.7 ± 3.5	17.3 ± 7.6
MJJG3	204.3 ± 1.3	11.4 ± 0.5	2232.6 ± 9.6	293.5 ± 3.8	1334.6 ± 9.8	722.8 ± 10.8	120.7 ± 44.3
MJJG4	126.6 ± 1	15.9 ± 0.9	1086.9 ± 8.6	136.2 ± 2.6	780.3 ± 4.7	368.2 ± 7.3	50.2 ± 22.3
MJJG5	114 ± 0.7	19.3 ± 0.3	931.4 ± 4.4	159.3 ± 2.6	677.5 ± 3.6	412 ± 18.9	77.1 ± 12

**FIGURE 8:**
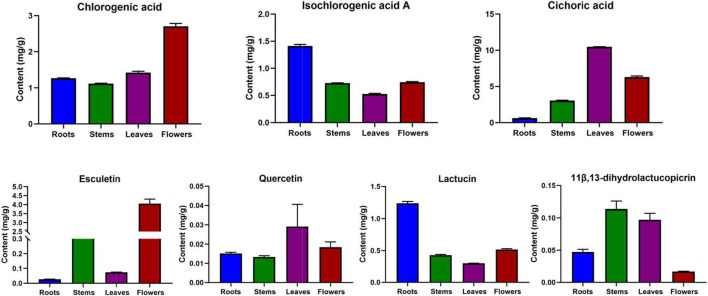
Distribution characteristics of the seven components in the roots, stems, leaves, and flowers of chicory.

During the previous research and sample collection, according to the market supply and the collection in the production area, we found that the main production area of chicory is the Xinjiang region and most of them are wild products. However, in view of the current environmental protection and desertification control, wild chicory is forbidden to be harvested, thus causing a shortage of medicinal resources. At the same time, our laboratory has conducted the cultivation of chicory medicinal herbs, and harvested chicory cultivars through the steps of land selection, breeding, transplanting, post-management, and harvesting. Therefore, this study also compared the differences in chemical composition of different parts of cultivated chicory (2-year-old) roots, stems, leaves, and flowers (Figure 8). In the flowers, chlorogenic acid and esculetin were higher in chicory flowers. A high content of chicoric acid and quercetin were found in the leaves. Isochlorogenic acid A and lactucin were relatively high in the roots. In the stems, a relatively high content of 11β,13- dihydrolactucopicrin was found. This trend was consistent with the trend observed in the large number of samples collected.

### Network Pharmacology and Molecular Docking Analysis of Dominant Components

**FIGURE 9:**
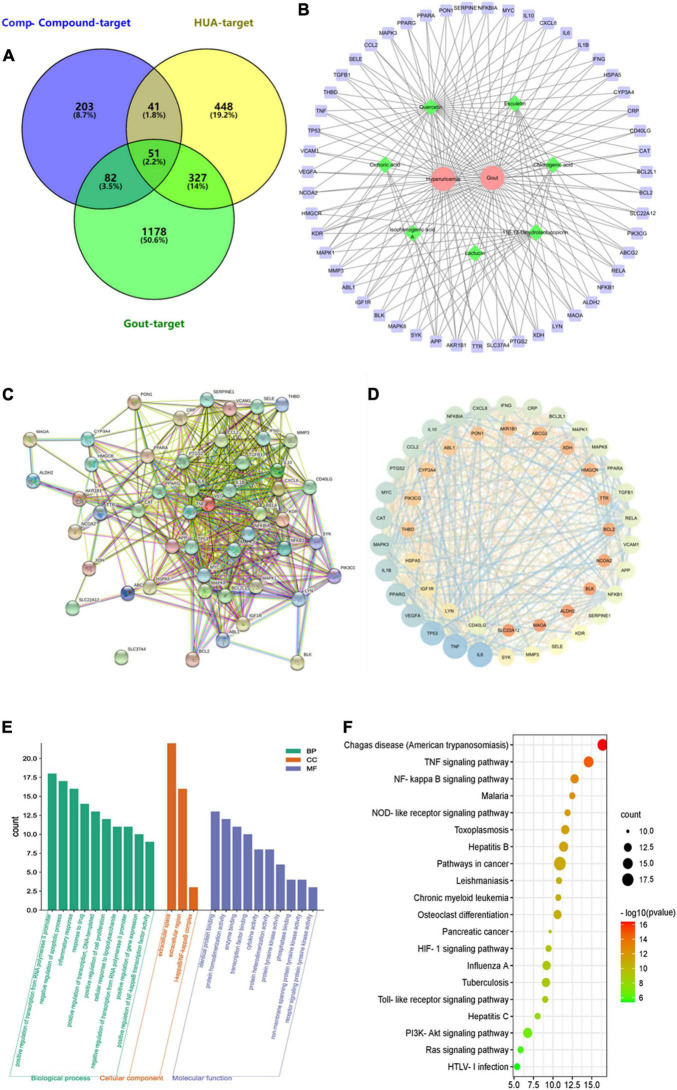
The target network of chicory. **(A)** The Venn diagram between constituent-related targets and gout-HUA-related targets; **(B)** Component-target-disease network. Red represents the disease, green represents the component, and purple represents the target; **(C)** The PPI network; **(D)** The PPI visual network diagram; **(E)** Component-disease target GO functional enrichment histogram; and **(F)** KEGG analysis.

Network pharmacology is often able to correlate and predict potential dominant ingredient targets of action and diseases. In this study, seven target components of chicory (chlorogenic acid, esculetin, lactucin, cichoric acid, isochlorogenic acid A, 11β,13-dihydrolactucopicrin, and quercetin) were searched in the Herb Herbology database and Swiss Target Prediction database, and 377 component targets were obtained after integrating and removing duplicates. The keywords “hyperuricemia, gout arthritis” were searched in OMIM, Drugbank, GeneCards, and DisGeNET databases, and 867 targets for hyperuricemia and 1,638 targets for gout were obtained after integrating and removing duplicates. The 377 component targets, 867 hyperuricemia disease targets, and 1,638 gout disease targets were imported into the Venny 2.1 online tool for Venn diagramming, and 51 component-disease common targets were obtained. As shown in Figure 9A. Seven target components and 51 component-disease targets in chicory were imported into Cytoscape 3.7.2 software, and the “component-disease-target” interaction network of chicory was drawn (Figure 9B).

The 51 key targets were imported into the STRING 11.5 database, and 51 nodes with 535 edges were obtained. The proteins with strong interactions were VEGF-KDR, TP53-BCL2L1, RELA-NFKBIA, etc. Among them, SLC37A4 had no interaction with other proteins (Figure 9C). Topological analysis was performed using Network Analyses (Figure 9D). Analysis using the CytoHubba tool yielded the core targets as IL-1β, TNF, IL-6, TP53, VEGFA, PPARG, MAPK3, MYC, CAT, and CCL2. Among them, the degrees of freedom of uric acid absorption and metabolism-related proteins XDH and ABCG2 were 9 and 8, respectively. This result indicates that the indicator components may also act by regulating both.

Fifty-one key targets were imported into the DAVID 6.8 database for GO and KEGG enrichment analysis. The GO analysis yielded a total of 90 GO entries, including 77 entries for biological processes, mainly involving regulation of RNA polymerase II promoter transcription, regulation of apoptosis and proliferation process, response to inflammation and drugs, immune response, protein phosphorylation, lipopolysaccharide-mediated signaling pathways, etc. The cellular component had three entries. The molecular function had 10 entries, mainly including protein binding, protein homodimer and heterodimer activity, enzyme binding, transcription factor binding, phosphatase binding, protein tyrosine kinase activity, etc. The top 10 entries of CC composition were selected with BP and MF entries (Figure 9E).

The KEGG enrichment analysis yielded a total of 82 entries, mainly enriched in pathways such as inflammatory response, signaling, and human diseases, involving TNF signaling pathway, NF-κB signaling pathway, NOD-like signaling pathway, TOLL-like signaling pathway, etc. The top 20 results were visualized and analyzed (Figure 9F).

**FIGURE 10:**
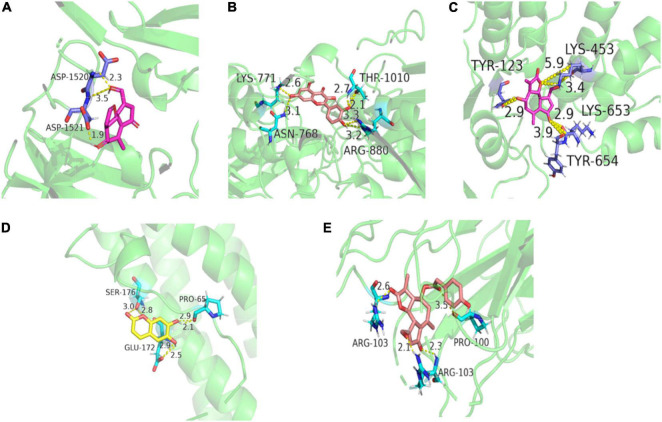
Molecular docking analysis. **(A)** Molecular docking result of lactucin with TP53; **(B)** molecular docking result of quercetin with XDH; **(C)** molecular docking result of lactucin with ABCG2; **(D)** molecular docking result of esculetin with IL-6; and **(E)** molecular docking result of 11β,13-dihydrolactucopicrin with TNF.

Molecular docking studies were performed to validate the biological activity of the seven key components. The 3D structures of IL-6 (PDB ID: 1ALU), TNF (PDB ID: 2E7A), TP53 (PDB ID: 6MXX), XDH (PDB ID: 1N5X), and ABCG2 (PDB ID: 6HBU) were downloaded from the PDB database, de-watered, de-liganded, and hydrogenated, and then docked with the Pubchem database obtained from the 3D structures of the seven indicator components. Each protein was processed with the component with the highest binding potential by visualization (the lowest binding energy ≤ −5.0 kJ/mol), and the results showed that quercetin and lactucin had a good binding activity with all five proteins (Figure 10).

### Biological Activity of Potential Markers

To further validate and identify the active ingredients in chicory based on chemical characterization and network pharmacological results, two cellular models were used in this study. Briefly, MSU-stimulated RAW264.7 cells to produce an inflammatory response as a brief model of gout to simulate the physiological pathogenesis of the disease. This was used as a gout model to evaluate the potential active compounds. On the other hand, L02 cells, using the liver as the target organ, were used to evaluate compounds with potential uric acid-lowering ability by administering xanthines to simulate the physiological process of uric acid production. Thus, the above two cellular models were applied for further screening and identification of chemically superior components in chicory.

**FIGURE 11:**
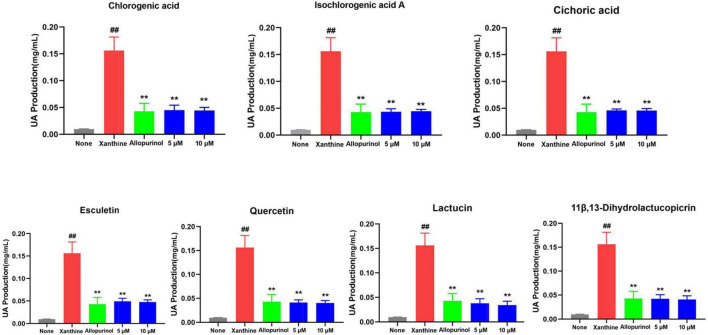
Seven compounds were able to significantly reduce uric acid production and their activity was comparable to that of allopurinol (*p* < 0.01, 5 μM, and 10 μM). The uric acid(UA)-lowering efficacy of seven compounds from chicory in L02 cells administered with xanthine. ##*p* < 0.01 vs None group; **p < 0.01 vs Xanthine group. *n* = 3, mean ± SD.

In humans, the pathogenesis of HUA and gout is dominated by the whole process from high uric acid to urate deposition to inflammatory outbreak. Thus, after confirming the appropriate concentration by CCK-8, chlorogenic acid, esculetin, lactucin, cichoric acid, isochlorogenic acid A, 11β,13-dihydrolactucopicrin, and quercetin in chicory were first validated for their potential inhibitory effect on uric acid production. As shown in Figure 11, seven compounds showed significance consistent uric acid production activity comparable to that of allopurinol (*p*<0.01, 5 μM, 10 μM). Allopurinol is a commonly used XOD inhibitor in clinical practice. It can inhibit the production of uric acid, thus achieving the therapeutic purpose of lowering uric acid. Here, we found that the superior components in chicory, represented by chicoric acid and lactucin, have good activity. Their mechanism of action may be related to the inhibition of XOD activity. This situation is consistent with the results of the network pharmacology evaluation. In this way, better XOD inhibitors contribute to discovering new drugs for the treatment of HUA and gout.

**FIGURE 12:**
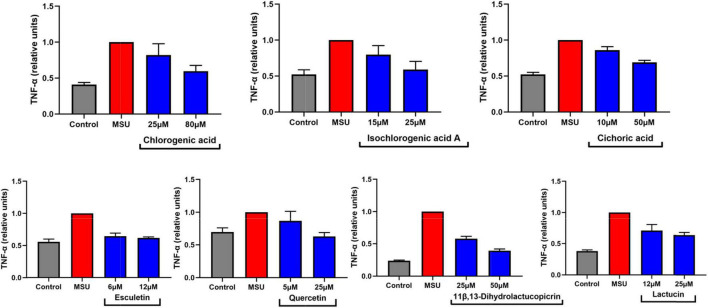
Seven compounds in chicory inhibits TNF-α secretion of RAW264.7 cells induced by MSU. The secretion of TNF-α was measured in the supernatant of RAW264.7 cells by ELISA. MSU, monosodium urate; TNF-α, tumor necrosis factor-α. *n* = 3, mean ± SD.

Tumor necrosis factor-α, a representative of inflammatory factors, has been reported in recent years to be closely associated with MSU-stimulated inflammation in gout disease. In this study, we found that TNF-α was associated with multiple pathways based on network pharmacology analysis. Therefore, we tried to stimulate the inflammatory response of RAW264.7 cells by MSU. Fortunately, we screened the dominant components of chicory and found that some of them have the potential ability to reduce the release of TNF-α by MSU-induced inflammation in macrophages (Figure 12). Although we did not see significant effects, we found a tendency for these components to reduce inflammatory factors. The reason for this may be due to the immaturity of this cell model. Therefore, in future studies, we intend to develop the optimization of this model in detail in order to better identify potential anti-inflammatory compounds in chicory. In fact, we could find that some of the components already released potential anti-inflammatory signals at low doses (5–25 μM). This also confirms the reported anti-inflammatory activity of chicory and its extracts.

### Recommended Quality Standards for Chicory

Based on the scientific connotation of Q markers in TCM, the quality control indexes in chicory should meet the requirements of Q markers in terms of potency, specificity, quality transferability, measurability, and compatibility (32). The results of content analysis of seven components, including chlorogenic acid, esculetin, lactucin, cichoric acid, isochlorogenic acid A, 11β,13- dihydrolactucopicrin, and quercetin, in the bulk samples showed that the content of components in different parts of chicory medicinal plants varied. Interestingly, we found that chicoric acid and lactucin were the two components with higher content in chicory. They are in the above-ground part and in the root, respectively. Compared with chlorogenic acid and esculetin, they have some composition specificity in chicory. Among them, lactucin can be used as a representative of sesquiterpene in chicory, and chicory acid can be used as a representative of polyphenolic compounds in chicory. Their contents are generally high, and the content trend is more stable. In addition, from the viewpoint of chromatographic analysis, the chromatographic separation of lactucin and chicory acid was non-interfering, and the separation was favorable. Therefore, they are the target compounds with compositional measurability properties in chicory.

For quality standards, when setting standard limits for chicory content, chicoric acid and lactucin are recommended as quality control indicators according to the results of this study. According to the current research on the chemical composition of chicory, chicoric acid and lactucin are the specific components of the genus Chicory in the family Asteraceae, so they can reflect the specificity and different characteristics of chicory. At present, chicoric acid has a legal reference, and the standard substance is available, and its stability and purity can meet the requirements of standard analysis.

For the limit indicators of different medicinal parts, in the root study, it was found that the chicory acid content in the roots originating from the main production area and the roots in the market differed greatly. In this study, the external standard method was used for the calculation of the content of all sample components. The content of the markers in the samples was calculated from the concentration of the corresponding reference standards. The content in roots from the main production area was able to be applied for limit setting, but the content in roots from the source market was generally low (0.02–0.05 mg/g), making it difficult to set consistent limits with the above-ground part. In contrast, lactucin was more stable in the above-ground part with the content in the roots. Thus, it is recommended to choose lactucin as the limit indicator for roots, and it can meet the mass transferability requirements. According to previous research (33, 34), chicoric acid and lactucin detection methods (HPLC) are beneficial to increase the implementation and operability of the chicory standard.

Furthermore, at the cellular level, pharmacodynamic experiments in this study showed that chicory acid and lactucin not only reduce uric acid production but also have the potential to inhibit the release of TNF-α inflammatory factors. They have therapeutic potential to control high uric acid and gout. Meanwhile, it is possible to correspond to the formula-evidence correspondence of chicory compounding, so that the quality research can return to the theory of TCM and reflect the expression of chicory effectiveness and the objective essence of its substances for diseases.

## Conclusion

In this study, a strategy to reveal bioactive-chemical quality markers was established. Through the comparative screening of fingerprint profiles of a large amount of *Cichorium glandulosum* Boiss.et Huet and *Cichorium intybus* L., superiority components were validated to be potential indicators of chemical quantitative properties for the roots and above-ground parts. Based on the content, bioinformatic analysis, and biological activity, chicory acid and lactucin were the main components that could reflect the anti-inflammatory and uric acid-lowering potential of chicory. The strategy contributes to the quality control, standard improvement, and rational clinical use of chicory.

## Data Availability Statement

The original contributions presented in this study are included in the article/Supplementary Material, further inquiries can be directed to the corresponding authors.

## Author Contributions

BZ conceived and designed the study. YL and SJ performed the experiments and analyzed the data. ZL, YW, HW, HJ, and SM supported the planning and interpretation of experiments. YL drafted the manuscript. All authors revised and approved the final manuscript.

## Conflict of Interest

The authors declare that the research was conducted in the absence of any commercial or financial relationships that could be construed as a potential conflict of interest.

## Publisher’s Note

All claims expressed in this article are solely those of the authors and do not necessarily represent those of their affiliated organizations, or those of the publisher, the editors and the reviewers. Any product that may be evaluated in this article, or claim that may be made by its manufacturer, is not guaranteed or endorsed by the publisher.

## Footnotes

1
http://herb.ac.cn/

2
http://www.swisstargetprediction.ch/

3
https://www.omim.org/

4
https://www.drugbank.ca/

5
http://www.genecards.org/

6
https://www.disgenet.org/

7
https://cn.string-db.org/cgi/input.pl

8
https://david.ncifcrf.gov/conversion.jsp

## Abbreviations

Q marker: Quality marker
TCM: traditional Chinese medicine
HPLC: high-performance liquid chromatography
LOD: limit of detection
LOQ: limit of quantification
CCK-8: Cell Counting Kit-8
DMSO: dimethyl sulfoxide
PBS: phosphate-buffered saline
KEGG: Kyoto Encyclopedia of Genes and Genomes
XOD: xanthine oxidase
ADA: adenosine deaminase
GLUT9: glucose transporter protein 9
HUA: hyperuricemia
MSU: monosodium urate
TNF-α: tumor necrosis factor-α.
