# Supplementary material for: Bioactive-Chemical Quality Markers Revealed: An Integrated Strategy for Quality Control of Chicory
Source: Front Nutr. 2022 Jul 4;9:934176. doi: 10.3389/fnut.2022.934176 (PMC9292578; doi:10.3389/fnut.2022.934176)
Supplement: Supplementary file 1 [file Data_Sheet_1.docx]

**SUPPLEMENTARY MATERIALS**

**Supplementary figures**


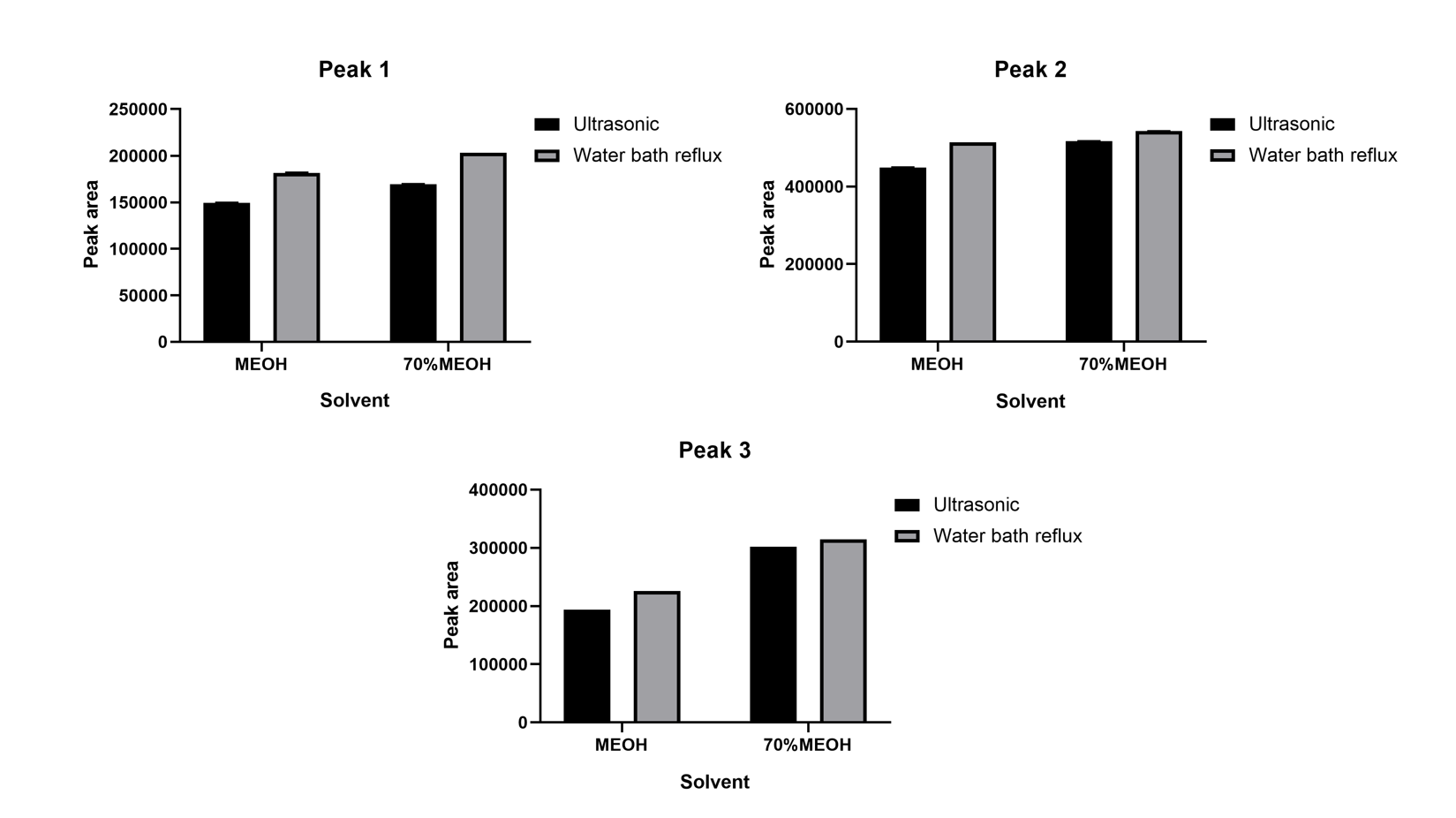


**FIGURE S1.** Results of comparison of different solvents and extraction methods.


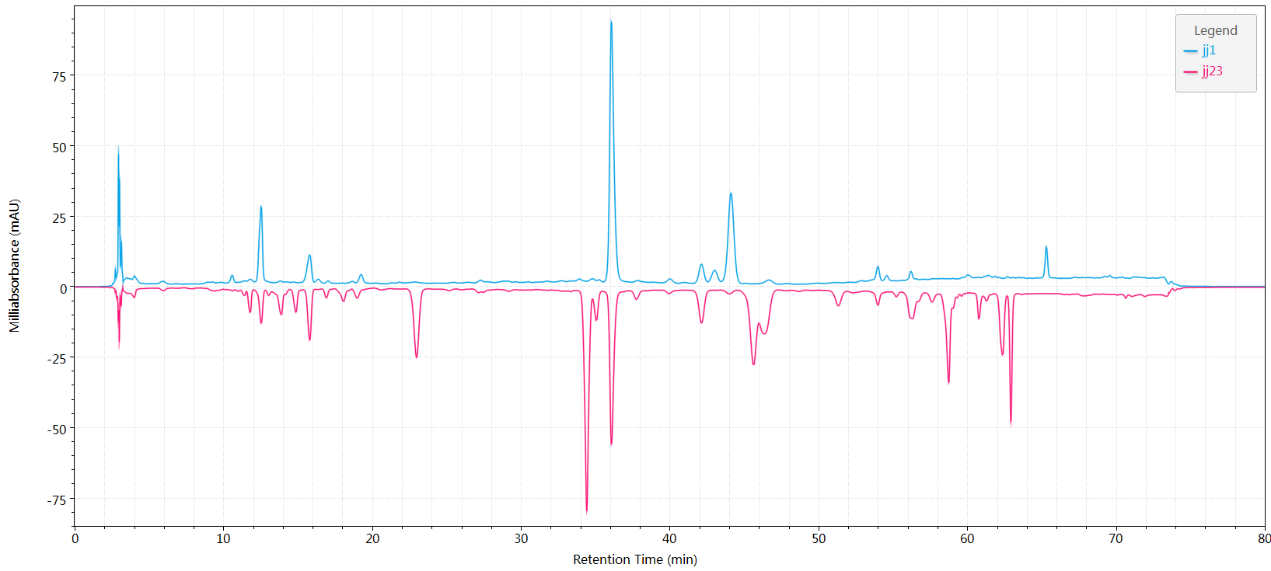


**FIGURE S2.** Mirror comparison chromatogram of leaves and stems in *Cichorium intybus* L.


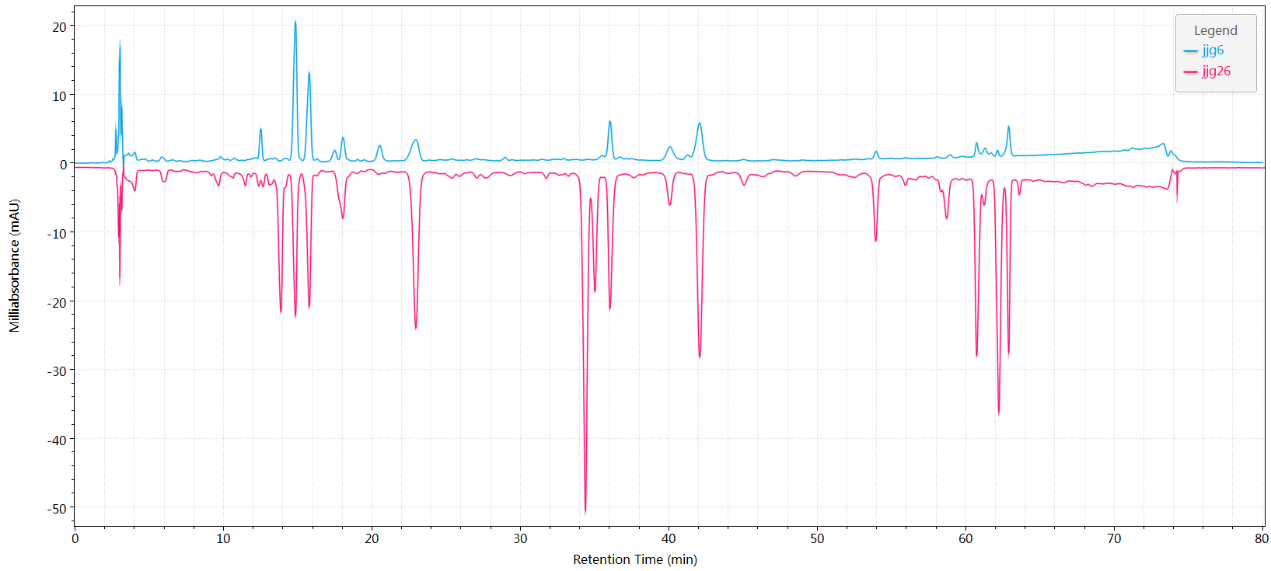


**FIGURE S3.** Mirror comparison chromatogram of cultivated and wild roots in *Cichorium intybus* L.


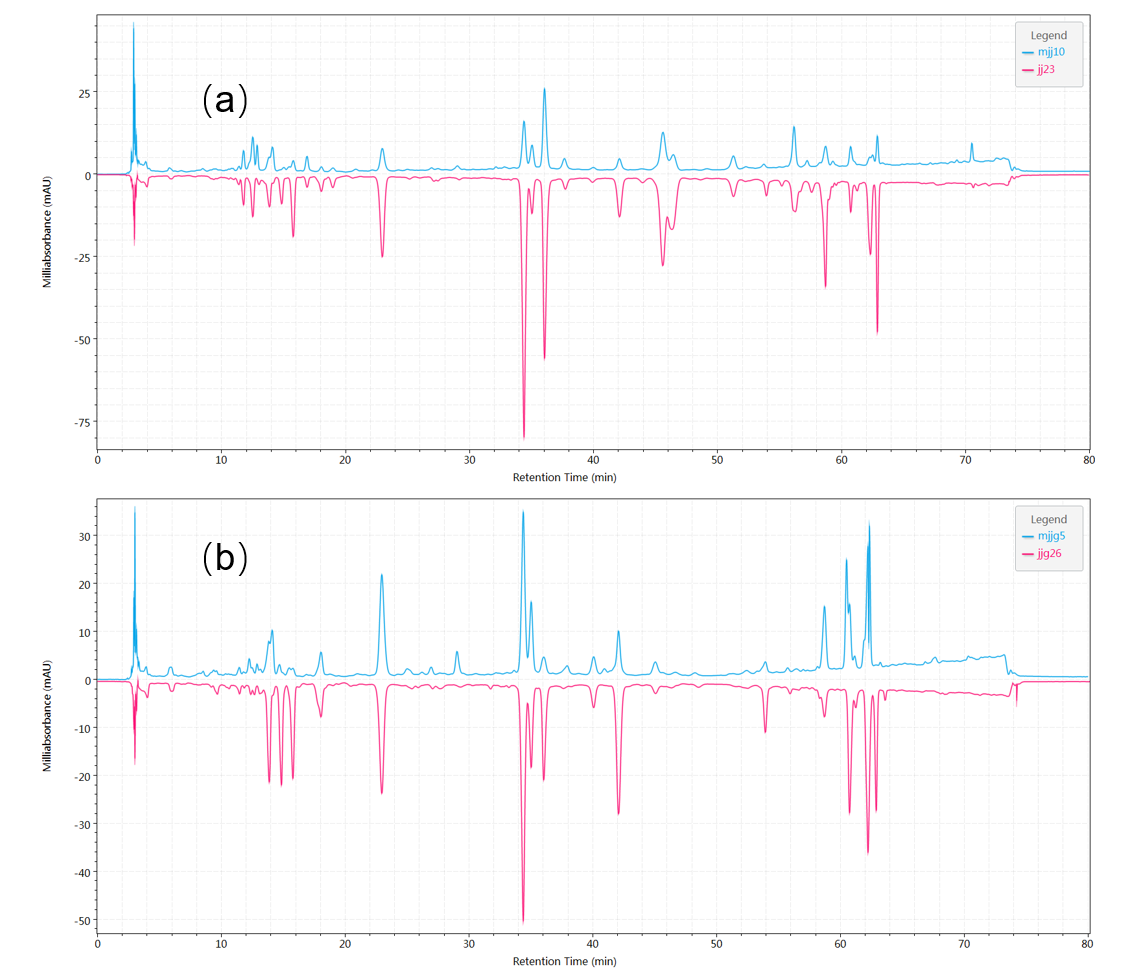


**FIGURE S4.** Mirror comparison chromatogram of above-ground parts (a) and roots (b) in *Cichorium intybus* L. and *Cichorium glandulosum* Boiss. et Huet.


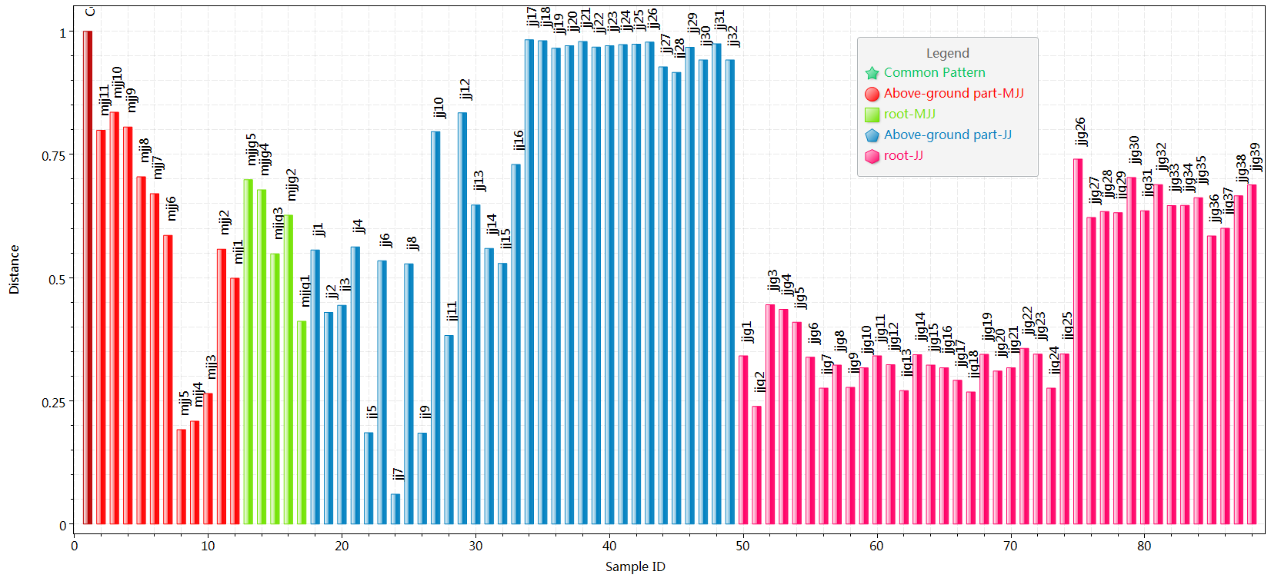


**FIGURE S5.** Similarity analysis of different medicinal parts (above-ground parts and roots) of Chicory.

**Supplementary tables**

**TABLE S1**. Sample collection information.

| Sample NO. | Species | Medicinal parts | Place of origin | Source of herbs | Collection Date |
| --- | --- | --- | --- | --- | --- |
| JJ1 | *Cichorium intybus* L. | Leaves | Liaoning | Anguo Herbal Market | 27/3/2021 |
| JJ2 | *Cichorium intybus* L. | Leaves | Hebei | Anguo Herbal Market | 26/3/2021 |
| JJ3 | *Cichorium intybus* L. | Leaves | Xinjiang | Anguo Herbal Market | 26/3/2021 |
| JJ4 | *Cichorium intybus* L. | Leaves | Anhui | Bozhou Herbal Market | 29/3/2021 |
| JJ5 | *Cichorium intybus* L. | Leaves | Guangxi | Bozhou Herbal Market | 28/3/2021 |
| JJ6 | *Cichorium intybus* L. | Leaves | Anhui | Anguo Herbal Market | 27/3/2021 |
| JJ7 | *Cichorium intybus* L. | Leaves | Guangxi | Bozhou Herbal Market | 28/3/2021 |
| JJ8 | *Cichorium intybus* L. | Leaves | Anhui | Bozhou Herbal Market | 28/3/2021 |
| JJ9 | *Cichorium intybus* L. | Leaves | Xinjiang | Shandong Chu Medicine Co. | 28/3/2021 |
| JJ10 | *Cichorium intybus* L. | Stems | Xinjiang | Xinjiang Minhai Traditional Chinese Medicine Beverage Co. | 26/3/2021 |
| JJ11 | *Cichorium intybus* L. | Stems | Xinjiang | Xinjiang Minhai Traditional Chinese Medicine Beverage Co. | 26/3/2021 |
| JJ12 | *Cichorium intybus* L. | Stems | Xinjiang | Xinjiang Minhai Traditional Chinese Medicine Beverage Co. | 26/3/2021 |
| JJ13 | *Cichorium intybus* L. | Stems | Hetian, Xinjiang | Xinjiang uygur autonomous region product quality supervision and inspection institute | 31/10/2020 |
| JJ14 | *Cichorium intybus* L. | Stems | Xinjiang | Xinjiang uygur autonomous region product quality supervision and inspection institute | 29/3/2021 |
| JJ15 | *Cichorium intybus* L. | Stems | Fukang, Xinjiang | Xinjiang uygur autonomous region product quality supervision and inspection institute | 31/10/2020 |
| JJ16 | *Cichorium intybus* L. | Stems | Hetian, Xinjiang | Xinjiang uygur autonomous region product quality supervision and inspection institute | 31/10/2020 |
| JJ17 | *Cichorium intybus* L. | Stems | Altai, Xinjiang | Xinjiang Minhai Traditional Chinese Medicine Beverage Co. | 1/8/2021 |
| JJ18 | *Cichorium intybus* L. | Stems | Altai, Xinjiang | Xinjiang Minhai Traditional Chinese Medicine Beverage Co. | 1/8/2021 |
| JJ19 | *Cichorium intybus* L. | Stems | Altai, Xinjiang | Xinjiang Minhai Traditional Chinese Medicine Beverage Co. | 1/8/2021 |
| JJ20 | *Cichorium intybus* L. | Stems | Altai, Xinjiang | Xinjiang Minhai Traditional Chinese Medicine Beverage Co. | 7/8/2021 |
| JJ21 | *Cichorium intybus* L. | Stems | Altai, Xinjiang | Xinjiang Minhai Traditional Chinese Medicine Beverage Co. | 7/8/2021 |
| JJ22 | *Cichorium intybus* L. | Stems | Altai, Xinjiang | Xinjiang Minhai Traditional Chinese Medicine Beverage Co. | 7/8/2021 |
| JJ23 | *Cichorium intybus* L. | Stems | Altai, Xinjiang | Xinjiang Minhai Traditional Chinese Medicine Beverage Co. | 7/8/2021 |
| JJ24 | *Cichorium intybus* L. | Stems | Altai, Xinjiang | Xinjiang Minhai Traditional Chinese Medicine Beverage Co. | 7/8/2021 |
| JJ25 | *Cichorium intybus* L. | Stems | Altai, Xinjiang | Xinjiang Minhai Traditional Chinese Medicine Beverage Co. | 7/8/2021 |
| JJ26 | *Cichorium intybus* L. | Stems | Altai, Xinjiang | Xinjiang Minhai Traditional Chinese Medicine Beverage Co. | 7/8/2021 |
| JJ27 | *Cichorium intybus* L. | Stems | Altai, Xinjiang | Xinjiang Minhai Traditional Chinese Medicine Beverage Co. | 7/8/2021 |
| JJ28 | *Cichorium intybus* L. | Stems | Altai, Xinjiang | Xinjiang Minhai Traditional Chinese Medicine Beverage Co. | 7/8/2021 |
| JJ29 | *Cichorium intybus* L. | Stems | Altai, Xinjiang | Xinjiang Minhai Traditional Chinese Medicine Beverage Co. | 7/8/2021 |
| JJ30 | *Cichorium intybus* L. | Stems | Urumqi, Xinjiang | Xinjiang uygur autonomous region product quality supervision and inspection institute | 3/4/2021 |
| JJ31 | *Cichorium intybus* L. | Stems | Yili, Xinjiang | Xinjiang uygur autonomous region product quality supervision and inspection institute | 13/8/2021 |
| JJ32 | *Cichorium intybus* L. | Stems | Altai, Xinjiang | Xinjiang Minhai Traditional Chinese Medicine Beverage Co. | 7/8/2021 |
| JJG1 | *Cichorium intybus* L. | Roots | Xinjiang | Anguo Herbal Market | 26/3/2021 |
| JJG2 | *Cichorium intybus* L. | Roots | Xinjiang | Anguo Herbal Market | 273/2021 |
| JJG3 | *Cichorium intybus* L. | Roots | Hotan, Xinjiang | Xinjiang uygur autonomous region product quality supervision and inspection institute | 31/12/2020 |
| JJG4 | *Cichorium intybus* L. | Roots | Jilin | Shenzhen Jiukang Yuan Biotechnology Co. | 27/3/2021 |
| JJG5 | *Cichorium intybus* L. | Roots | Unknown | Laboratory Retention Samples | 26/3/2021 |
| JJG-6 | *Cichorium intybus* L. | Roots | Unknown | Laboratory Retention Samples | 26/3/2021 |
| JJG7 | *Cichorium intybus* L. | Roots | Unknown | Bozhou Herbal Market | 26/3/2021 |
| JJG8 | *Cichorium intybus* L. | Roots | Xinjiang | Tong Ren Tang Co. | 24/3/2021 |
| JJG9 | *Cichorium intybus* L. | Roots | Hebei | Bozhou Herbal Market | 29/3/2021 |
| JJG10 | *Cichorium intybus* L. | Roots | Unknown | Anguo Herbal Market | 6/4/2021 |
| JJG11 | *Cichorium intybus* L. | Roots | Xinjiang | Anhui Daoyuantang Traditional Chinese Medicine Pill Co. | 24/3/2021 |
| JJG12 | *Cichorium intybus* L. | Roots | Xinjiang | Shandong Chu Medicine Co. | 28/3/2021 |
| JJG13 | *Cichorium intybus* L. | Roots | Unknown | Anguo Herbal Market | 5/4/2021 |
| JJG14 | *Cichorium intybus* L. | Roots | Xinjiang | Bozhou Herbal Market | 6/4/2021 |
| JJG15 | *Cichorium intybus* L. | Roots | Anhui | Anguo Herbal Market | 3/4/2021 |
| JJG16 | *Cichorium intybus* L. | Roots | Unknown | Bozhou Herbal Market | 29/3/2021 |
| JJG17 | *Cichorium intybus* L. | Roots | Jilin | Bozhou Herbal Market | 4/4/2021 |
| JJG18 | *Cichorium intybus* L. | Roots | Unknown | Anguo Herbal Market | 6/4/2021 |
| JJG19 | *Cichorium intybus* L. | Roots | Jilin | Anguo Herbal Market | 4/4/2021 |
| JJG20 | *Cichorium intybus* L. | Roots | Jilin | Anguo Herbal Market | 5/4/2021 |
| JJG21 | *Cichorium intybus* L. | Roots | Yunnan | Kunming Lushiwan Market | 5/4/2021 |
| JJG22 | *Cichorium intybus* L. | Roots | Xinjiang | Bozhou Herbal Market | 6/4/2021 |
| JJG23 | *Cichorium intybus* L. | Roots | Jilin | Anguo Herbal Market | 5/4/2021 |
| JJG24 | *Cichorium intybus* L. | Roots | Anhui | Bozhou Herbal Market | 4/4/2021 |
| JJG25 | *Cichorium intybus* L. | Roots | Xinjiang | Xinjiang uygur autonomous region product quality supervision and inspection institute | 4/4/2021 |
| JJG26 | *Cichorium intybus* L. | Roots | Altai, Xinjiang | Xinjiang Minhai Traditional Chinese Medicine Beverage Co. | 1/8/2021 |
| JJG27 | *Cichorium intybus* L. | Roots | Altai, Xinjiang | Xinjiang Minhai Traditional Chinese Medicine Beverage Co. | 7/8/2021 |
| JJG28 | *Cichorium intybus* L. | Roots | Altai, Xinjiang | Xinjiang Minhai Traditional Chinese Medicine Beverage Co. | 7/8/2021 |
| JJG29 | *Cichorium intybus* L. | Roots | Altai, Xinjiang | Xinjiang Minhai Traditional Chinese Medicine Beverage Co. | 7/8/2021 |
| JJG30 | *Cichorium intybus* L. | Roots | Altai, Xinjiang | Xinjiang Minhai Traditional Chinese Medicine Beverage Co. | 7/8/2021 |
| JJG31 | *Cichorium intybus* L. | Roots | Altai, Xinjiang | Xinjiang Minhai Traditional Chinese Medicine Beverage Co. | 7/8/2021 |
| JJG32 | *Cichorium intybus* L. | Roots | Altai, Xinjiang | Xinjiang Minhai Traditional Chinese Medicine Beverage Co. | 7/8/2021 |
| JJG33 | *Cichorium intybus* L. | Roots | Altai, Xinjiang | Xinjiang Minhai Traditional Chinese Medicine Beverage Co. | 7/8/2021 |
| JJG34 | *Cichorium intybus* L. | Roots | Altai, Xinjiang | Xinjiang Minhai Traditional Chinese Medicine Beverage Co. | 7/8/2021 |
| JJG35 | *Cichorium intybus* L. | Roots | Altai, Xinjiang | Xinjiang Minhai Traditional Chinese Medicine Beverage Co. | 7/8/2021 |
| JJG36 | *Cichorium intybus* L. | Roots | Altai, Xinjiang | Xinjiang Minhai Traditional Chinese Medicine Beverage Co. | 7/8/2021 |
| JJG37 | *Cichorium intybus* L. | Roots | Urumqi, Xinjiang | Xinjiang uygur autonomous region product quality supervision and inspection institute | 3/4/2021 |
| JJG38 | *Cichorium intybus* L. | Roots | Yili, Xinjiang | Xinjiang uygur autonomous region product quality supervision and inspection institute | 13/8/2021 |
| JJG39 | *Cichorium intybus* L. | Roots | Altai, Xinjiang | Xinjiang Minhai Traditional Chinese Medicine Beverage Co. | 7/8/2021 |
| MJJ1 | *Cichorium glandulosum* Boiss.et Huet | Stems | Urumqi, Xinjiang | Xinjiang uygur autonomous region product quality supervision and inspection institute | 26/3/2021 |
| MJJ2 | *Cichorium glandulosum* Boiss.et Huet | Stems | Hotan, Xinjiang | Xinjiang Minhai Traditional Chinese Medicine Beverage Co. | 7/8/2021 |
| MJJ3 | *Cichorium glandulosum* Boiss.et Huet | Stems | Urumqi, Xinjiang | Xinjiang uygur autonomous region product quality supervision and inspection institute | 1/9/2021 |
| MJJ4 | *Cichorium glandulosum* Boiss.et Huet | Stems | Urumqi, Xinjiang | Xinjiang uygur autonomous region product quality supervision and inspection institute | 2/9/2021 |
| MJJ5 | *Cichorium glandulosum* Boiss.et Huet | Stems | Urumqi, Xinjiang | Xinjiang uygur autonomous region product quality supervision and inspection institute | 3/9/2021 |
| MJJ6 | *Cichorium glandulosum* Boiss.et Huet | Stems | Urumqi, Xinjiang | National Institutes for Food and Drug Control | 3/3/2004 |
| MJJ7 | *Cichorium glandulosum* Boiss.et Huet | Stems | Urumqi, Xinjiang | National Institutes for Food and Drug Control | 1/3/2021 |
| MJJ8 | *Cichorium glandulosum* Boiss.et Huet | Stems | Laboratory cultivars | | 11/9/2021 |
| MJJ9 | *Cichorium glandulosum* Boiss.et Huet | Stems | Laboratory cultivars | | 11/9/2021 |
| MJJ10 | *Cichorium glandulosum* Boiss.et Huet | Stems | Laboratory cultivars | | 11/9/2021 |
| MJJ11 | *Cichorium glandulosum* Boiss.et Huet | Stems | Laboratory cultivars | | 11/9/2021 |
| MJJG1 | *Cichorium glandulosum* Boiss.et Huet | Roots | Hotan, Xinjiang | Xinjiang uygur autonomous region product quality supervision and inspection institute | 7/8/2021 |
| MJJG2 | *Cichorium glandulosum* Boiss.et Huet | Roots | Laboratory cultivars | | 11/9/2021 |
| MJJG3 | *Cichorium glandulosum* Boiss.et Huet | Roots | Laboratory cultivars | | 11/9/2021 |
| MJJG4 | *Cichorium glandulosum* Boiss.et Huet | Roots | Laboratory cultivars | | 11/9/2021 |
| MJJG5 | *Cichorium glandulosum* Boiss.et Huet | Roots | Laboratory cultivars | | 11/9/2021 |
